# Supplementary material for: Association of lifestyle and behavioral factors with self-reported visual problems among schoolchildren in rural Bangladesh
Source: PLoS One. 2025 Aug 21;20(8):e0328541. doi: 10.1371/journal.pone.0328541 (PMC12370125; doi:10.1371/journal.pone.0328541)
Supplement: Attachment — Submitted filename: Revision letter R1_Editorial comments.docx [file pone.0328541.s003.docx]

PONE-D-25-00919

Association of lifestyle and behavioral factors with self-reported visual problems among schoolchildren in rural Bangladesh.

Dear Editor,

Thank you for your positive feedback.

Please see my responses in BLUE.

Best regards

Fakir M Amirul Islam

A/Professor, School of Health Science

Swinburne University of Technology.

Editorial comments:

1. A rebuttal letter that responds to each point raised by the academic editor and reviewer(s). You should upload this letter as a separate file labeled 'Response to Reviewers'.

Response: The manuscript is now formatted according to PLOS One guidelines

2.	Please provide a complete Data Availability Statement in the submission form, ensuring you include all necessary access information or a reason for why you are unable to make your data freely accessible. If your research concerns only data provided within your submission, please write "All data are in the manuscript and/or supporting information files" as your Data Availability Statement.

Response: Data are now available.

Data S1 HLS EU_PLOS One.xlsx

3. We note that the grant information you provided in the ‘Funding Information’ and ‘Financial Disclosure’ sections do not match.

Response: I have made this correction.

Please address in detail the comments from reviewer #2. In particular, please carefully consider the comments related to limitations of the sampling methods, as well as suggestions regarding the statistical analyses. Please clarify the statistical analyses and results.

Response: I have carefully checked and addressed the feedback.
